# Optimization of Fermentation Process of *Zanthoxylum bungeanum Seeds* and Evaluation of Acute Toxicity of Protein Extract in Mice

**DOI:** 10.3390/foods13244004

**Published:** 2024-12-11

**Authors:** Xueyan Gu, Guowei Xu, Chunhua Liang, Raktham Mektrirat, Lei Wang, Kang Zhang, Bingbing Meng, Xi Tang, Xiaoya Wang, Hanyurwumutima Egide, Jiahui Liu, Haiyu Chen, Mingxi Zhang, Jingyan Zhang, Xuezhi Wang, Jianxi Li

**Affiliations:** 1 Chinese-Thai Traditional Chinese Veterinary Medicine and Techniques Cooperation Laboratory, Lanzhou Institute of Husbandry and Pharmaceutical Sciences, Chinese Academy of Agricultural Sciences, Lanzhou 730050, China; 15803685475@163.com (X.G.); guoweixu1158@126.com (G.X.); liang835216@163.com (C.L.); wanglei807@126.com (L.W.); 15719319192@163.com (K.Z.); mengbingbing2000@126.com (B.M.); syyxuan_040304@163.com (X.T.); wangxy9625@163.com (X.W.); egide.bhanyurwumutima@isabu.bi (H.E.); 13608696436@163.com (J.L.); 13473301560@163.com (H.C.); 18531607688@163.com (M.Z.); jianxil@163.com (J.L.); 2 Chinese-Thai Traditional Chinese Veterinary Medicine and Techniques Cooperation Laboratory, Faculty of Veterinary Medicine, Chiang Mai University, Chiang Mai 50200, Thailand; raktham.m@cmu.ac.th; 3 Lanzhou Veterinary Research lnstitute, Chinese Academy of Agricultural Sciences, Lanzhou 730030, China

**Keywords:** *Bacillus subtilis*, *Lactobacillus plantarum*, solid state fermentation, *Zanthoxylum bungeanum seeds*, security, fermented *Zanthoxylum bungeanum seeds* protein

## Abstract

The seeds of *Zanthoxylum bungeanum seeds*, a high-quality vegetable protein source, encounter application limitations due to their high molecular weight and anti-nutritional factors. This study focused on optimizing the fermentation process by investigating key parameters such as inoculation amount, inoculation ratio, material-to-liquid ratio, fermentation temperature, and fermentation time. Both single-factor experiments and response surface methodology were used to determine the optimal conditions. The effects of fermentation on particle size, surface morphology (scanning electron microscopy), water holding capacity, oil holding capacity, solubility, and emulsification properties of *Zanthoxylum bungeanum seed* protein were analyzed. In addition, acute toxicity was investigated at doses of 1.5 g/kg, 3 g/kg, 6 g/kg, and 12 g/kg. The results showed that the optimal fermentation conditions were an inoculum concentration of 10%, a ratio of *Bacillus subtilis* to *Lactobacillus plantarum* of 1:1, a material-to-liquid ratio of 0.8:1, a temperature of 35 °C, and a fermentation period of 4 days. Under these optimized conditions, the soluble protein content reached 153.1 mg/g. After fermentation, the functional properties of *Zanthoxylum bungeanum seed* protein improved significantly: the water holding capacity increased by 89%, the oil holding capacity by 68%, while the emulsifying activity and stability indices improved by 6% and 17%, respectively. The macromolecular proteins in the seeds of *Zanthoxylum bungeanum* were effectively broken down into smaller fragments during fermentation, resulting in a more folded and porous surface structure. In acute toxicity tests, all mice treated with fermented *Zanthoxum seed* protein survived for more than 7 days after injection, and there were no significant differences in body weight, organ index, and hematological tests between groups, but FZBSP of 1.5 g/kg~12 g/kg caused varying degrees of steatosis and inflammatory damage in the heart and liver. In conclusion, this study confirms that follow-up pilot studies using 1.5 g/kg FZBSP have the potential for further development and utilization.

## 1. Introduction

*Zanthoxylum bungeanum seeds* (ZBS) are one of the most important by-products of *Zanthoxylum bungeanum*, with a protein content of about 14–20% (based on *Zanthoxylum bungeanum* cultivars), which can reach more than 60% after defatting [1]. It is rich in amino acids and contains a variety of essential amino acids for the human body. In addition, it is easily absorbed and has a high nutritional value [2,3]. According to statistics, China produces about 1.1 million tons of ZBS every year [4]. In the food industry, ZBS are currently used as raw materials for the development of a variety of foods, including condiments [5], flavored snacks, and special beverages [6]. In medicine and health, ZBS have demonstrated antioxidant and anti-tumor activities [7] and cholesterol-lowering effects [8,9]. In agriculture, ZBS have been shown to improve the growth performance of livestock and poultry and at the same time reduce feed costs [10]. In industry, it can be used to produce insecticides and building materials with anti-corrosive properties [11,12]. These products are usually used as a primary processed product to replace some conventional feeds in the field of livestock and poultry breeding, thereby reducing the production costs of the breeding industry [13]. However, research and development of ZBS as highly processed products in the food and feed sectors is still limited. Studies have found that probiotics are dominant in fermented feed. Their metabolites can reduce feed pH [14], decrease harmful microorganisms [15,16], inhibit mycotoxin production, eliminate allergens and anti-nutritional factors [17], and enhance the crude protein content of feed [18]. There are significant quality differences among different fermentation processes. Currently, enhancing the nutritional value of feed through probiotic fermentation and the synergy between bacteria and enzymes has become a hot topic in new feed industry research [19,20].

Solid-state fermentation (SSF) is a common method for feed production, which is characterized by its low cost and mild reaction conditions. During the fermentation process, SSF can effectively remove harmful elements in feed, convert macromolecular proteins into low molecular weight proteins, and release free amino acids [21]. Common fermentative probiotics include *Bacillus*, lactic acid bacteria, and yeasts [22]. Among these *Bacillus subtilis* (*B. subtilis*) strains and *Lactobacillus plantarum* (*L. plantarum*) strains are common fermentation strains [23,24], which are characterized by rapid growth, relatively low nutrient requirements, rapid secretion of large amounts of proteins and metabolites, and no production of toxins [25]. The fermentation of functional hemp milk by *B. subtilis* can enhance its nutritional value and functional properties [26]. Fermentation by *L. plantarum* can improve the solubility, adsorption capacity, and paste viscosity of millet starch [26]. Co-fermentation feed with *B. subtilis* and *L. plantarum* can significantly improve the intestinal flora and strengthen the immunity of the animals [27].

It has been reported that probiotics capable of bioconverting alkylamides and alkaloids were isolated and identified from Zanthoxylum seed meal [28]. This research aimed to compare and analyze the effects of co-fermentation using *B. subtilis* and *L. plantarum* on the nutrient composition of ZBS. The objective is to explore multiple applications for ZBS in food, feed, environmental protection, agriculture, and other fields, thereby enhancing its comprehensive application value and economic benefits across various sectors [10,29]. The aim of this study was to characterize the changes in the fermented ZBS protein and to evaluate its physicochemical properties, including water holding capacity, oil holding capacity, and particle size distribution. We also investigated the safety of the fermented ZBS protein by performing acute toxicity tests in mice. The cumulative results of these studies pave the way for various potential applications of fermented ZBS protein in food, feed, environmental protection, and agriculture, thereby improving its utility and economic viability in various fields.

## 2. Materials and Methods

### 2.1. Materials and Reagents

The number of effective viable *B. subtilis* was ≥1.0 × 10^10^ CFU/g, and the number of effective viable *L. plantarum* was ≥1.0 × 10^10^ CFU/g. These were screened and stored by the Lanzhou Institute of Husbandry and Pharmaceutical Chinese Academy of Agricultural Sciences. The patent of *B. subtilis* and *L. plantarum* is No. ATCC6633 and No. CICC20871. The ZBS were collected in the planting base of the professional agricultural cooperative of *Zanthoxylum bungeanum* planting in Liangshui Town, Wudu District, Gansu Province, and the test ZBS were the grafted *Zanthoxylum bungeanum* of local “Dahongpao” *Zanthoxylum bungeanum*. ZBS refers to the ripe Zanthoxum seeds extracted from the peel of Zanthoxum after harvesting [30]. These are crushed (25,000 2 min) using a universal high-speed crusher (China solar ball TYQ-1000g, Guangzhou, China), passed through an 80-mesh sieve, and then stored in a refrigerator at −20 °C. The Bradford assay kit was purchased from Beijing Boxsun Technology Co., Ltd. (Beijing, China). The 2,2-diphenyl-1-picrylhydrazyl radical scavenging capacity (DPPH RSC) assay kit, the ABTS radical scavenging capacity (ABTS RSC) assay kit, and the hydroxyl radical scavenging capacity (HRSC) assay kit, respectively, following the manufacturer’s instructions. All assay kits were procured from Solarbio (Beijing, China). MRS medium and NB medium were purchased from Guangdong Huankai Biological Technology Co., Ltd. (Guangzhou, China). The complex protease (S10155, 120 U/g) was obtained from Shanghai Yuanye Bio-Technology Co., Ltd. (Shanghai, China). All other reagents used were of analytical grade.

### 2.2. Experimental Animals

Healthy SPF mice (body weight, 20–22 g; age 4–6 weeks). All experimental animals used in this study were obtained from the Lanzhou Veterinary Research Institute, Chinese Academy of Agricultural Sciences. The mice were adaptively fed for 5–7 days prior to the start of the experiment to minimize the effects of environmental stressors. The mice were then randomly assigned to different groups of six animals each, and the sawdust was replaced every 24 h. The mice had access to water AD libitum before and throughout the experiment. The animals were handled and euthanized according to internationally accepted guidelines. The research was licensed by the ethical approval of the Lanzhou Institute of Husbandry and Pharmaceutical Sciences of the Chinese Academy of Agricultural Science (Statement No. AEC-CAAS-20231124, Lanzhou, China).

### 2.3. Activation and Expansion of Strains

*L. plantarum* and *B. subtilis* stored at −80 °C were retrieved and dissolved in a water bath at 37 °C. *L. plantarum* was inoculated in MRS Broth, and *B. subtilis* in NB broth, then incubated at 37 °C with shaking at 120 rpm for 24 h to obtain the primary seed liquid. Using a 2% inoculum, the first-generation seed liquid was added to the corresponding broth medium for passage. The third-generation probiotics were then used as fermentation seed liquid for subsequent experiments.

### 2.4. Solid State Fermentation

Accurately weigh 100.0 g ZBS powder, 0.2 g ammonium sulfate, and 1 g glucose and add them into a 500 mL fermentation bottle to prepare SSF medium. A total of 8% of the amount of *B. subtilis* and *L. plantarum* inoculum liquids were added to the SSF medium, and the inoculum amount and ZBS were mixed thoroughly for fermentation.

### 2.5. Single Factor Experiment

According to the procedure described in 2.4, 100.0 g of Zanthoxylum seed powder was weighed and fermented at a fermentation temperature of 35 °C for 2 days, with an inoculation ratio of *B. subtilis* and *L. plantarum* of 1:1. The inoculations amount of *B. subtilis* and *L. plantarum* (6%, 8%, 10%, 12%, and 14%) were studied. Measured by substrate mass), inoculation proportion (1:1, 1:2, 2:1, 2:3, and 3:2), material–liquid ratio (0.6:1, 0.7:1, 0.8:1, 0.9:1, and 1:1), fermentation temperature (30 °C, 33 °C, 35 °C, 37 °C, and 40 °C), and fermentation time (0, 2, 4, 6, and 8 d). The specific experimental conditions of each factor are shown in Table 1. Only one variable was changed for each experiment, and the crude protein content at each factor level was used to determine the response surface test range. All tests were performed in triplicate according to LiW [31].

### 2.6. Response Surface Optimization Experiment

Based on the results of the above single-factor experiments, response surface optimization tests were conducted. The inoculation amount (A), the material–liquid ratio (B), the fermentation temperature (C), and the fermentation time (D) were employed as independent variables, with the protein yield serving as the response value. Following the Box–Behnken experimental design, the fermented ZBS protein content was utilized as the response. The application’s response surface test can offer empirical relationships between the independent variables and the response variables based on parameter estimation [32], aiming to determine the optimal extraction process. The design of the response surface experimental factor level is presented in Table 2.

### 2.7. Protein Extraction from Fermented ZBS and ZBS

Based on the optimal fermentation conditions obtained by the above tests, the method of reference [33] was modified. Fermentation ZBS and ZBS (2 g), 5.43% complex protease (based on substrate mass), and phosphate buffer (2 g/50 mL) were placed in an Erlenmeyer flask and then extracted at 51.3 °C for 3 h. The supernatant was collected by centrifugation and precipitated with hydrochloric acid (1 mol/L). The precipitation was then centrifuged, collected, and dissolved with NaOH (1 mol/L), and the pH was adjusted to 7. The collected solution was freeze-dried to obtain fermented *Zanthoxylum bungeanum* seeds protein (FZBSP) or Zanthoxylum bungeanum seeds protein (ZBSP) or stored at −20 °C.

### 2.8. Structure Analysis

#### 2.8.1. Particle Size Analysis

The ZBSP and FZBSP were dissolved in sterile water at a concentration of 1 mg/mL. This procedure was conducted following the method outlined by YILDIZ [34], with appropriate adjustments made to suit our specific needs. Subsequently, the samples were analyzed using a Mastersizer 3000 laser particle size analyzer to determine their particle size distribution.

#### 2.8.2. Scanning Electron Microscope

Following the method described by DOU [35] and others, images of FZBSP and ZBSP structures were scanned. The FZBSP and ZBSP were placed on an aluminum plate coated with gold powder through sputtering. SEM images of the FZBSP and ZBSP were obtained using field emission SEM (JSM-840, JEOL, Tokyo, Japan).

### 2.9. Physicochemical Properties

#### 2.9.1. Solubility

The method was adapted from MIR [36] and others with slight modifications. TFZBSP and ZBSP (100 mg) were stirred with distilled water (90 mL) at 25 °C for 1 h and then centrifuged (10,000 rpm, 30 min) to collect the supernatant. Mix the supernatant with 1 X Coomassie brilliant blue solution. Absorbance was measured at 595 nm using bovine serum albumin as the standard. The formula for calculating the solubility of a protein sample is as follows:

$$S_{0}=N_{S}/N_{t}\times 100\%.$$

Type: *S*_0_ for solubility; *N_s_* for the amount of protein in the supernatant; and *N_t_* total is the amount of protein in the sample.

#### 2.9.2. Water Holding Capacity

The methods reference Zhang [37] and others, with slight modifications. A 500 mg sample of FZBSP or ZBSP was weighed into a centrifuge tube, and 10 mL of distilled water was added to measure water holding capacity. The mixture was vortexed and shaken for 5 min, followed by centrifugation at 3050 g for 10 min. After centrifugation, the unbound water on the upper layer was poured off. The formula to calculate the water-holding capacity (WHC) of the protein is as follows:

$$WHC\left(\%\right)=(a-b)/c\times 100\%,$$

where a is the weight of the centrifuge tube after dumping supernatant (g), b is the weight of the centrifuge tube and protein (g), and c is protein weight (g).

#### 2.9.3. Oil Holding Capacity

Refer to the method described by Liu [38] et al., with a slight modification. A total of 500 mg of FZBSP or ZBSP samples were weighed into a 50 mL centrifuge tube, 10 g of soybean oil was added, vortexized for 30 s, placed at 37 °C for 24 h, and centrifuged at 4000 rpm for 20 min. Weighed after removing the upper oil, the protein oil holding capacity (OHC) is calculated as follows:

$$OHC\left(\%\right)=(a-b)/c\times 100\%,$$

where a is the weight of the centrifuge tube after dumping supernatant (g), b is the weight of the centrifuge tube and protein (g), and c is protein weight (g).

#### 2.9.4. Emulsion Activity and Emulsion Stability

The method was adapted from Chen [39] et al., with slight modifications. To 1 mg/mL of FZBSP or ZBSP, 8 mL of solution mixed with 2 mL of soybean oil, homogeneous under an automatic homogenate machine (16,000 r/min, 1 min). Homogeneous in 0 min and 10 min after the completion of emulsion and 0.1% from the bottom of the drain, 100 mL + SDS solution diluted 50 times, 500 nm absorbance value, after blending with 0.1% SDS solution as the blank. The emulsifying activity index (EAI, m^2^/g) and emulsification stability (ESI, min) computation formulas are as follows:

$$EAI=(2\times 2.303\times A_{0}\times N)/(C\times \left(1-\theta \right)\times 10,000,$$

$$ESI=A_{0}/(A_{0}-A_{10})\times 10.$$

Type: A_0_, 0 min absorbance value; A_10_, 10 min absorbance value; N, diluted times, 50; C, protein concentration, g/mL; and θ, oil phase volume fraction, 0.2.

### 2.10. Antioxidant Properties

To compare the antioxidant properties of FZBSP and ZBSP components, refer to Sittiruk’s [40] method, modified slightly. DPPH RSC, ABTS RSC, and HRSC were measured according to the instructions, and OD values were measured at 515 nm, 405 nm, and 536 nm, respectively. In the determination process, FZBSP was compared with ZBSP to prepare a 5 mg/mL solution, and all tests were performed 3 times.

### 2.11. Assessment of Acute Toxicity

Referring to Raskosha’s [41] method, sixty C57BL/6 mice were randomly divided into 5 groups with 12 mice in each group and were fed adaptively for 1 week in the control group and FZBSP group (1.5, 3, 6, 12 g/kg), respectively. The mice were fasted for 12 h before administration, given water and freedom, and the other groups were given the same dose of FZBSP except the blank group, which was given normal saline. Then, the appearance, signs, and adverse reactions of mice were observed for 7 consecutive days. At the end of the 7th day, blood was collected through the orbital vein, then the neck was removed and blood samples were collected for hematological and blood biochemical analysis. Then, the mice were surgically dissected, the lesions of the major internal organs were visually examined, the number of tissue organs and organ index were recorded, and the following organs were histologically analyzed: heart, liver, spleen, lung, and kidney.

## 3. Statistical Analysis

SPSS 26.0 was used to process the data. The response surface design was performed using Design Expert 11.0.4. Chart mapping was completed using GraphPad Prism 8. All experimental results are presented as “mean ± standard deviation”, with significant differences considered at *p* < 0.05 and no significant difference at *p* > 0.05.

## 4. Result and Analysis

### 4.1. Optimization of Fermentation Conditions

#### 4.1.1. Single-Factor Analysis

**Figure 1:**
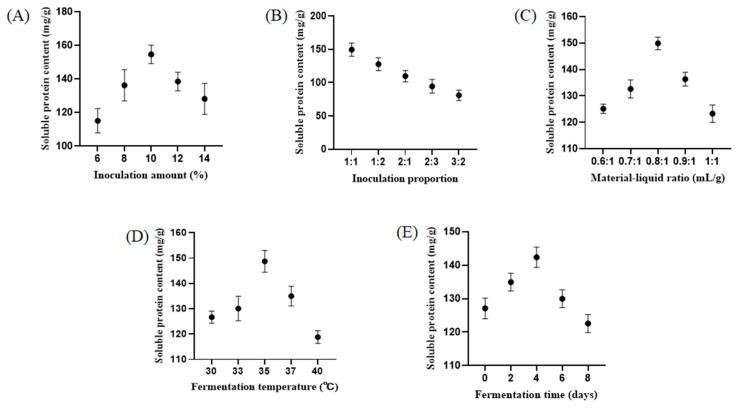
Inoculation amount (**A**), inoculation proportion (**B**), material–liquid ratio (**C**), fermentation temperature (**D**), and fermentation time (**E**) on the yield of soluble protein content of FZBS. All tests were performed 3 times. Each data point represents the mean ± SD of triplicates.

To investigate the effects of different conditions on fermented ZBS, five sub-experiments were established for a single-factor experiment to determine the optimal conditions based on soluble protein content in the fermentation broth. As shown in Figure 1, the maximum soluble protein content in the fermentation broth was achieved at a strain inoculation amount of 10%. Similar trends were observed in the experiments in which the fermentation temperature, fermentation time, and material–liquid ratio were varied, and the maximum soluble protein content in the fermentation broth was obtained at an inoculation ratio of 1:1, a material–liquid ratio of 0.8:1, a fermentation temperature of 35 °C, and a fermentation time of 4 d.

#### 4.1.2. Box–Behnken Design Results

The Box–Behnken design was implemented using Design-Expert 11.0.4., as shown in Table 2, Table 3 and Table 4. Fermented ZBS soluble protein was considered as the response value, with strain inoculation amount, material–liquid ratio, fermentation temperature, and fermentation time as the four factors for the experimental design. Quadratic polynomial regression analysis was performed on the experimental data, resulting in the equation

$$Y=151.9-2.023\times A-4.17\times B-0.0231\times C+2.30\times D+5.71\times AB+5.98\times AC+1.17\times AD+\;1.51\times BC+1.58\times BD-7.19\times CD-12.50\times A^{2}-5.80\times B^{2}-14.26\times C^{2}-14.42\times D^{2}$$

Y represents the soluble protein contents, while A, B, C, and D correspond to bacterial inoculum, material–liquid ratio, fermentation temperature, and fermentation time. This model’s coefficient of determination R^2^ is 0.9576, and the adjusted R^2^ (Adj. R^2^) is 0.9152. The model’s *p*-value was found to be less than 0.0001, indicating a strong correlation between the predicted and actual values. The results showed that B, D, AB, AC, CD, A2, B2, C2, and D2 have remarkable effects on the extraction yield of fermented ZBS soluble protein (*p* < 0.01). Furthermore, the *p*-values for AD, BC, and BD interactions were greater than 0.05, suggesting no significant interaction between these pairs of factors. This analysis demonstrates the effectiveness of the model in predicting and evaluating the influence of each factor on protein content.

**Figure 2:**
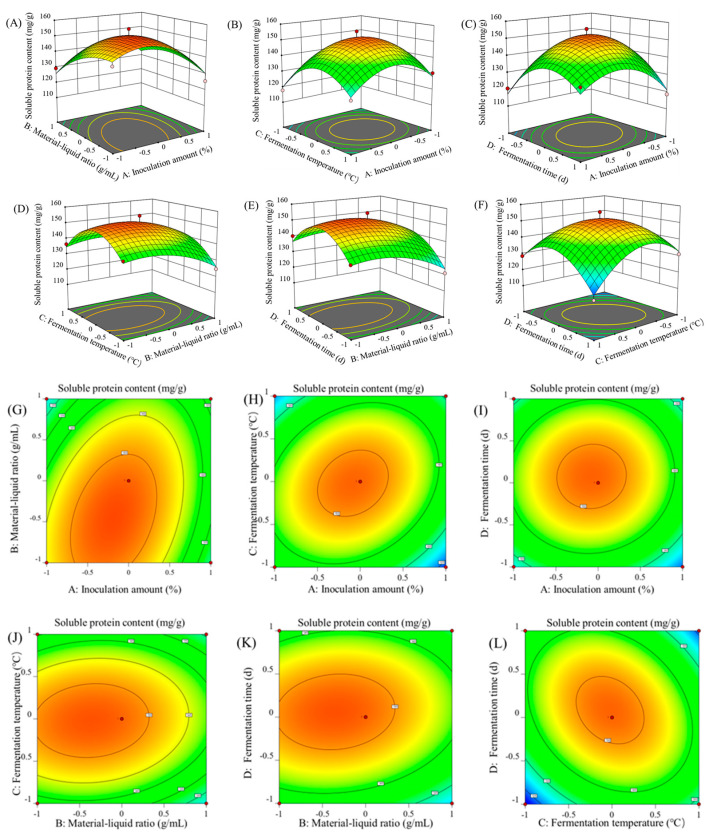
Response surface plots (**A**–**F**) and the corresponding contour plots (**G**–**L**) showing inoculation amount and material–liquid ratio (**A**,**G**); inoculation amount and fermentation temperature (**B**,**H**); inoculation amount and fermentation time (**C**,**I**); material–liquid ratio and fermentation temperature (**D**,**J**); material–liquid ratio and fermentation time (**E**,**K**); and fermentation temperature and fermentation time (**F**,**L**).

As shown in Figure 2, the response surface curve and contour map were generated to analyze the impact of inoculum amount, material–liquid ratio, fermentation temperature, and fermentation time on soluble protein content. The optimal fermentation conditions predicted by the model were 11.5% inoculum volume, a 0.78:1 (g/mL) ratio of solid to liquid, a 35.13 °C fermentation temperature, and a 3.75 d fermentation time. According to the experimental conditions, the optimal fermentation conditions were determined as the inoculated amount of the strain of 10%, the solid–liquid ratio of 0.8:1 (g/mL), the fermentation temperature of 35 °C, and the fermentation time of 4 d. The soluble protein content could reach 153.1 (mg/g), which is in close agreement with the predicted value and shows that the model can well reflect the actual situation of optimizing the mixed fermentation process of *B. subtilis* and *L. plantarum* on ZBS.

### 4.2. The Structural Characteristics of ZBSP and FZBSP

#### 4.2.1. Particle Size Distribution

**Figure 3:**
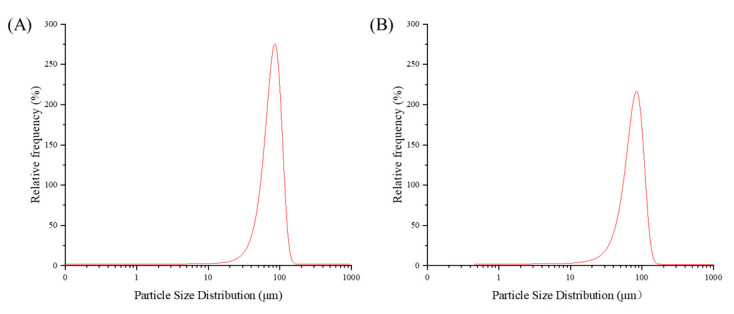
The particle size distribution of ZBSP (**A**) and FZBSP (**B**). All tests were performed 3 times.

As shown in Figure 3, the alterations in particle size and distribution of FZBSP and ZBSP were illustrated. The particle size of ZBSP was about 275.22 μm, while the particle number of FZBSP was significantly reduced to about 216.33 μm. After fermentation, the particle size of FZBSP was reduced by 27.2% compared with that of ZBSP.

#### 4.2.2. Scanning Electron Microscopy

**Figure 4:**
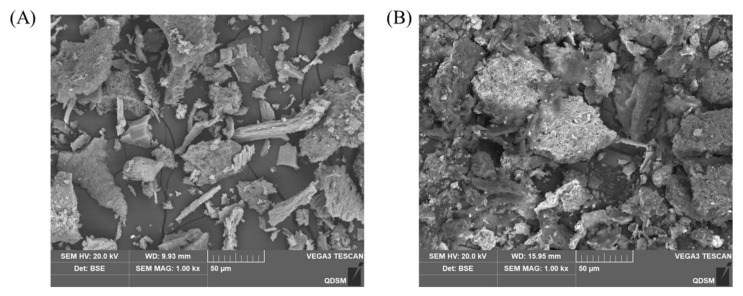
Scanning Electron Microscopy of ZBSP (**A**) and FZBSP (**B**). All tests were performed 3 times.

As Figure 4 shows, ZBSP exhibited a flat layered structure on the surface under the electron microscope, with the larger particles having smooth edges. In contrast, FZBSP exhibited a more fragmented and irregular layered structure, and the surface of the particles became wrinkled and porous due to the co-fermentation of *B. subtilis* and *L. plantarum*. After fermentation, the protein structure was looser, indicating that the mixed fermentation of *B. subtilis* and *L. plantarum* could completely decompose the protein material, which was more conducive to digestion and absorption and also improved the nutritional properties of ZBSP.

### 4.3. The Physicochemical Properties of ZBSP and FZBSP

**Figure 5:**
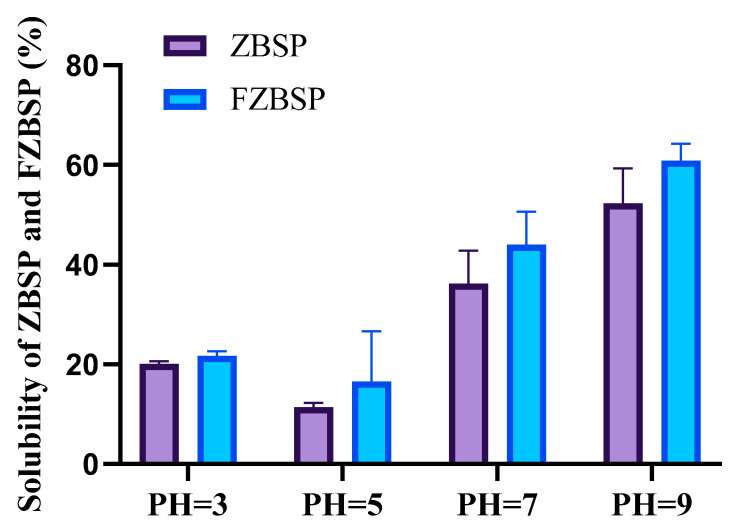
When pH = 3, pH = 5, pH = 7, pH = 39, the solubility of ZBSP and FZBSP. Results are expressed as mean ± SD (*n* = 3). ZBSP means *Zanthoxylum bungeanum seeds* protein, and FZBSP means fermented *Zanthoxylum bungeanum seeds* protein.

As shown in Figure 5, the solubility of ZBSP before and after fermentation was low at pH 3, which is close to the isoelectric point, but showed an upward trend with the increase of pH from 5 to 9. At pH 5 and pH 7, the solubility of peppercorn seed protein after fermentation was 45.34% and 21.72% higher than before fermentation, respectively. At pH 9, the solubility of ZBSP and FZBSP was 52.28% and 60.85%, respectively. According to Table 5, the WHC and OHC of FZBSP were 3.87 ± 0.11 (g/g) and 6.16 ± 0.19 (g/g), respectively, and those of ZBSP were 1.99 ± 0.10 (g/g) and 3.07 ± 0.01 (g/g), respectively. EAI and ESI of FZBSP were 16.22 ± 0.17 (m^2^/g) and 54.61 ± 2.41 (min), respectively, and those of ZBSP were 15.26 ± 0.29 (m^2^/g) and 46.82 ± 1.64 (m^2^/g), respectively. After fermentation, WHC, OHC, EAI, and ESI of FZBSP were significantly increased (*p* < 0.01).

### 4.4. Antioxidant Activity

According to Table 6, the antioxidant activity of ZBSP was significantly improved after fermentation with probiotics. The DPPH RSC, ABTS RSC, and HRSC in FZBSP were 63.85%, 95.42%, and 65.39%, respectively, which were higher than those of ZBSP.

### 4.5. Acute Toxicity Test

#### 4.5.1. Effect on General Condition

After intragastric administration, all mice in each dose group survived and showed normal behavior with food and water intake, good mental status, no signs of irritability or excessive startle response, and other abnormalities.

#### 4.5.2. Effect on Body Weight

**Figure 6:**
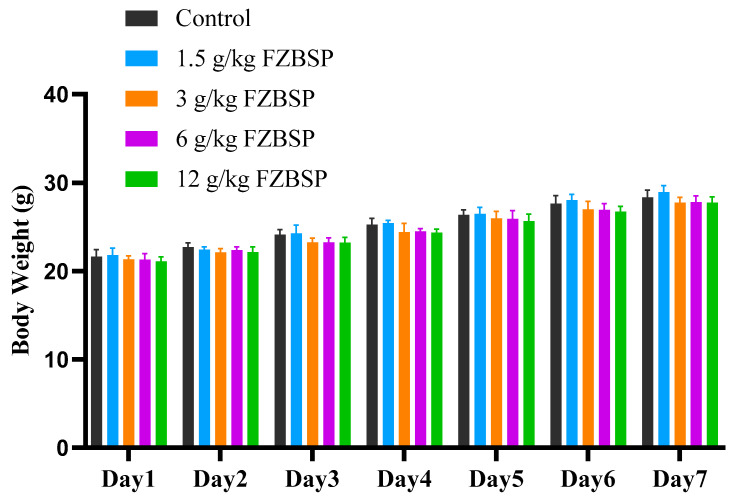
Impact on body weight of mice in FZBSP toxicity. Six mice were randomly selected in each group.

As shown in Figure 6, the mice in the 1.5 g/kg, 3 g/kg, 6 g/kg, and 12 g/kg dose groups showed no significant changes in body weight after 7 days of feeding compared to the blank group (*p* > 0.05), but a steady increase in body weight was observed throughout the study period. The organ indices of the 1.5 g/kg, 3 g/kg, 6 g/kg, and 12 g/kg dose groups were compared with those of the CON group. As shown in Table 7, there was no significant difference in the organ index between the groups (*p* > 0.05).

#### 4.5.3. Routine Blood Tests

As shown in Table 8, the blood parameters (WBC, LYM, MON, NEU, RBC, HGB, and MCV) in the 1.5 g/kg, 3 g/kg, 6 g/kg, and 12 g/kg dose groups compared with the CON group all showed abnormal changes attributable to drug administration. No significant difference (*p* > 0.05) was observed.

#### 4.5.4. Serum Biochemical Tests

According to Table 9, compared with the CON group, no significant changes were observed in ALP, ALT, BUN, GLU, and TP levels when fed FZBSP at 1.5 g/kg, 3 g/kg, 6 g/kg, and 12 g/kg compared with the CON group, indicating no obvious toxic effects on liver function and renal function.

#### 4.5.5. Pathological Section

**Figure 7:**
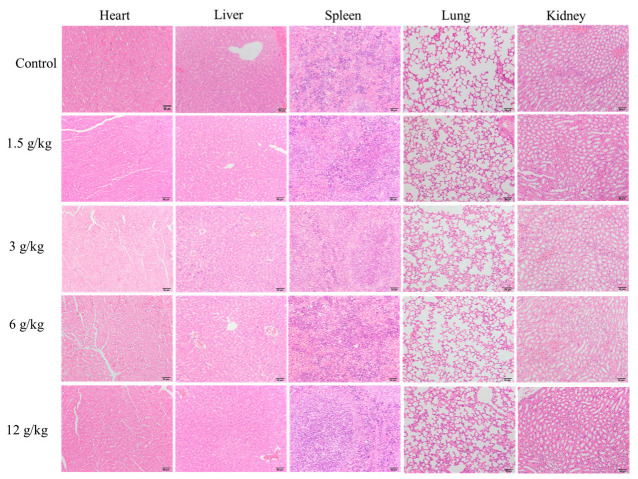
Impact on histopathological examination of mice in FZBSP toxicity.

Figure 7 shows the pathological staining sections of heart, liver, spleen, lung, and kidney tissues of mice from the CON group and the 1.5 g/kg~12 g/kg FZBSP dose groups. The study shows that the structural integrity of spleen, kidney, and lung tissue sections in the control group and all drug-treated groups is well preserved with clear structures; no significant inflammatory cell infiltration was observed. Consistent with the findings in the CON group. Steatosis and inflammatory infiltration were observed in the heart and liver in the FZBSP dose groups compared to the CON group.

## 5. Discussion

In recent years, probiotic fermentation has become increasingly important as a means of supporting the extraction of active plant ingredients [42]. At the same time, solid-state fermentation is being used in many industries to improve the nutritional quality and functional properties of agricultural by-products [43]. It has been found that the co-fermentation of *Lactobacillus plantarum* and *Bacillus subtilis* can effectively increase the solubility of TCA protein content in rice bran [44], promote direct absorption by the gastrointestinal tract, and induce antioxidant and immunoregulatory functions [45]. Fermented soybean meal can increase the crude protein content and lactate content of soybean meal by 50.1% and 94.9%, respectively [46]. The physical and chemical properties of roasted soybean flour can be changed [47]. In this study, the effects of inoculation amount, inoculation ratio, solid–liquid ratio, fermentation temperature, and fermentation time on FZBSP during mixed fermentation of *Bacillus subtilis* and *Lactobacillus plantarum* were evaluated. The results showed that as the inoculation amount, fermentation temperature, and time were increased, the FZBSP content first increased and then decreased. The optimum fermentation conditions for the extraction of FZBSP from ZBS were an inoculation amount of 10%, an inoculation ratio of 1:1, a solid–liquid ratio of 0.8:1, a fermentation temperature of 35 °C, and a fermentation time of 4 d. The technological fermentation technology obtained in this study provides a theoretical basis for the further development and utilization of ZBS. 

The combination of scanning electron microscopy and particle size analysis showed that FZBSP has a rough surface and consists of small particles. This observation can be related to the protein-degrading enzymes produced during the fermentation process [48]. Furthermore, the reduction in particle size of FZBSP suggests that FZBSP undergoes more thorough hydrolysis, resulting in the formation of protein structures with smaller molecules. Compared to ZBSP, the protein properties of FZBSP are significantly improved. In particular, the water holding capacity is increased by 89%, the oil holding capacity by 68%, and the EAI and ESI are increased by 6% and 17%, respectively. These improvements may be attributed to increased protein charge and intermolecular electrostatic repulsion, increased protein surface area, reduced size, and inhibition of protein aggregation facilitated by fermentation [49]. Protein solubility is considered the most practical indicator of the functional properties of proteins. Adequate solubility is a fundamental prerequisite for effective protein expression and emulsification. Consequently, improving protein solubility can improve the overall performance of protein emulsification [50,51,52]. Oil-holding capacity indicates the ability of a protein to bind with free fatty acids. A higher oil-holding capacity indicates a stronger ability of the protein to bind oil, thus minimizing oil loss during production and improving food flavor [53]. Due to their amphiphilic properties, proteins can act as emulsifiers and increase the stability of emulsions by inhibiting processes such as emulsification, coalescence, precipitation, and flocculation of droplets [54]. Antioxidant defense serves as a universal mechanism in the body [55,56], capable of repairing and preventing oxidative damage [57]. During the fermentation process, compounds present in ZBSP undergo biotransformation, resulting in the production of compounds with varied antioxidant properties [58]. Following fermentation, there is an increase in DPPH RSC clearance by 8%, ABTS RSC clearance by 7%, and HRSC clearance by 22.5%. These increases indicate that fermentation enhances antioxidant performance, consistent with the findings of Wu et al. in their study [59].

In this study, no deaths or abnormal reactions were observed in mice within 7 days after a high dose (12 g/kg) of FZBSP. According to the World Health Organization standards for acute toxicity of exogenous chemicals, the FZBSP used in this study is considered safe, non-toxic, and well tolerated. Blood plays a crucial role in maintaining the homeostasis of the animal’s internal environment, so relevant serum indicators can objectively reflect the health status of the body [60,61]. Compared with the control group, there were no significant changes in the serum levels of ALP, ALT, BUN, GLU, and TP in each dose group of FZBSP. However, FZBSP caused steatosis and inflammatory injury of the heart and liver and had no adverse effects on the spleen, kidney function, or lungs. In addition, we found no statistically significant changes in the mice’s organ weight or organ-to-body ratio. Therefore, combined with the results of blood analysis and pathological sections, the FZBSP (1.5 g/kg) dose is recommended for subsequent experimental studies.

## 6. Conclusions

The optimal fermentation conditions for ZBS using a mixed culture of *B. subtilis* and *L. plantarum* were beneficial for increasing the FZBSP content. Compared to ZBSP, particle size analysis and SEM images show that FZBSP has a more porous structure on the surface, with protein particles degrading on the surface. In terms of protein properties, FZBSP exhibited increased solubility, water binding, oil binding, and emulsifiability, favoring digestion and absorption. The acute toxicity test of FZBSP on mice showed that the growth performance, blood analysis, and pathological sections of the spleen, lung, and kidney of mice had no significant changes at the dose of FZBSP, but the heart and liver showed varying degrees of steatosis and inflammatory damage. Considering the changes in protein structure and properties and the results of the acute toxicity test in mice, we can summarize that the nutritional quality of ZBSP after solid-phase fermentation with *B. subtilis* and *L. plantarum* is optimized, its nutritional value is enhanced, and a dose of 1.5 g/kg is recommended for subsequent relevant experimental studies. This study paves the way for a re-evaluation of FZBSP as a potential functional food ingredient.

## Figures and Tables

**Table 1:** Single-factor test design.

Single Factor	Fixed Factors
Inoculation Amount (6%, 8%, 10%, 12%, and 14%)	1:1, 0.7:1 g/mL, 35 °C, 2 d
Inoculation Proportion (1:1, 1:2, 2:1, 2:3, and 3:2)	10%, 0.7:1 g/mL, 35 °C, 2 d
Material–Liquid Ratio (0.6:1, 0.7:1, 0.8:1, 0.9:1, and 1:1 g/mL)	10%, 1:1, 35 °C, 2 d
Fermentation Temperature (30, 33, 35, 37, and 40 °C)	10%, 1:1, 0.8:1 g/mL, 2 d
Fermentation Time (d) (0, 2, 4, 6, and 8 d)	10%, 1:1, 0.8:1 g/mL, 35 °C

**Table 2:** Factors and levels in the response surface analysis.

Factor	Symbol Code	Levels		
		−1	0	1
Inoculation Amount (%)	A	8	10	12
Material–Liquid Ratio (g/mL)	B	1:0.6	1:0.8	1:1
Temperature (°C)	C	33	35	37
Time (d)	D	2	4	6

**Table 3:** Experimental data of the four-factor, three-level Box–Behnken design for study response.

Runs	Inoculation Amount (%)	Material–Liquid Ratio (g/mL)	Temperature (°C)	Time (Days)	Soluble Protein Contents (mg/g)
1	0	0	0	0	150.87
2	0	1	0	−1	120.97
3	−1	−1	0	0	143.18
4	0	0	−1	−1	115.24
5	0	−1	−1	0	138.8
6	0	1	0	1	131.78
7	0	1	−1	0	124.78
8	0	−1	0	−1	135.6
9	−1	0	1	0	118.63
10	−1	1	0	0	129.24
11	0	−1	0	1	140.11
12	1	0	1	0	132.75
13	−1	0	−1	0	130.38
14	0	−1	1	0	136.43
15	−1	0	0	1	130.38
16	0	1	1	0	128.45
17	0	0	−1	1	131.08
18	−1	0	0	−1	128.08
19	0	0	0	0	150.87
20	1	0	−1	0	120.56
21	0	0	0	0	155.96
22	0	0	0	0	151.16
23	1	1	0	0	134.33
24	1	−1	0	0	125.42
25	1	0	0	−1	117.78
26	0	0	1	−1	128.6
27	1	0	0	1	124.77
28	0	0	0	0	150.87
29	0	0	1	1	115.7

**Table 4:** ANOVA with the response face quadratic model.

Source	Sum of Squares	DF	Mean Square	F Value	*p*-Value	Significance
Model	3503.64	14	250.26	22.58	<0.0001	***
A—Inoculation Amount	49.12	1	49.11	4.43	0.0538	
B—Liquid–Material Ratio	208.22	1	208.22	18.79	0.0007	**
C—Temperature	0.0064	1	0.0064	0.0006	0.9812	
D—Time	63.26	1	63.26	5.71	0.0315	*
AB	130.54	1	130.54	11.78	0.004	**
AC	143.21	1	143.21	12.92	0.0029	**
AD	5.48	1	5.48	0.4947	0.4934	
BC	9.1	1	9.1	0.8211	0.3802	
BD	9.94	1	9.94	0.897	0.3597	
CD	206.53	1	206.53	18.63	0.0007	**
A^2^	1013.06	1	1013.06	91.41	<0.0001	***
B^2^	218.07	1	218.07	19.68	0.0006	**
C^2^	1318.8	1	1318	118.99	<0.0001	***
D^2^	1349.22	1	1349.22	121.74	<0.0001	***
Residual	155.17	14	11.08			
Lack of Fit	134.95	10	13.49	2.67	0.1783	not significant
Pure Error	20.22	4	5.05			
Cor Total	3658.81	28				

Note: R^2^ = 0.9576; Predicted R^2^ = 0.7789; Adjusted R^2^ = 0.9152; Adequate Precision = 15.9291. *: *p* < 0.05, **: *p* < 0.01, ***: *p* < 0.001.

**Table 5:** Physicochemical properties of ZBSP and FZBSP.

Items	ZBSP	FZBSP	*p*-Value
WHC (g/g)	1.99 ± 0.10 ^b^	3.87 ± 0.11 ^a^	<0.001
OHC (g/g)	3.07 ± 0.01 ^b^	6.16 ± 0.19 ^a^	<0.001
EAI (m^2^/g)	15.26 ± 0.29 ^b^	16.22 ± 0.17 ^a^	<0.05
ESI (min)	46.82 ± 1.64 ^b^	54.61 ± 2.41 ^a^	<0.05

Note: Results are expressed as mean ± SD (*n* = 3). Different letters indicate significant differences in the same line (*p* < 0.05). ZBSP means *Zanthoxylum bungeanum seeds* protein, and FZBSP means fermentation *Zanthoxylum bungeanum seeds protein*.

**Table 6:** Antioxidant activity of FZBSP and ZBSP (%).

Items	ZBSP	FZBSP	*p*-Value
DPPH RSC	58.80 ± 0.02 ^b^	63.85 ± 0.01 ^a^	<0.05
ABTS RSC	89.34 ± 0.02 ^b^	95.42 ± 0.02 ^a^	<0.05
HRSC	53.38 ± 0.01 ^b^	65.39 ± 0.01 ^a^	<0.001

Note: Results are expressed as mean ± SD (*n* = 3). Different letters indicate significant differences in the same line (*p* < 0.05). DPPH RSC means 2,2-diphenyl-1-picrylhydrazyl radical scavenging capacity; ABTS RSC means ABTS radical scavenging capacity; HRSC means hydroxyl radical scavenging capacity; ZBSP means *Zanthoxylum bungeanum seeds* protein; and FZBSP means fermented *Zanthoxylum bungeanum seeds protein*.

**Table 7:** Effect of FZBSP on organ index in mice.

Items	Heart	Liver	Spleen	Lungs	Kidney
CON	5.04 ± 0.26	45.48 ± 4.11	3.46 ± 0.74	6.64 ± 1.15	10.97 ± 1.11
1.5 g/kg	5.31 ± 0.34	46.31 ± 5.31	3.37 ± 0.72	6.66 ± 1.33	10.91 ± 1.05
3 g/kg	5.25 ± 0.26	44.67 ± 3.91	3.44 ± 0.60	6.91 ± 0.57	9.96 ± 1.37
6 g/kg	5.25 ± 0.69	44.16 ± 1.61	3.69 ± 0.47	6.85 ± 1.01	10.41 ± 1.19
12 g/kg	5.68 ± 0.71	45.50 ± 2.88	3.75 ± 0.71	6.94 ± 1.20	10.60 ± 1.33

Note: Results are expressed as mean ± SD (*n* = 6).

**Table 8:** Hematological data of the toxicity study of the FZBSP.

Groups	WBC (10^9^/L)	LYM (%)	MON (%)	NEU (%)	RBC (10^12^/L)	HGB (g/dL)	MCV (fL)
CON	4.05 ± 0.73	3.58 ± 0.65	0.23 ± 0.06	0.58 ± 0.13	10.07 ± 0.26	15.18 ± 0.47	48.33 ± 1.97
1.5 g/kg	3.96 ± 0.29	3.11 ± 0.32	0.17 ± 0.06	0.68 ± 0.12	9.11 ± 0.79	13.60 ± 1.37	47.83 ± 2.71
3 g/kg	4.04 ± 0.53	3.46 ± 0.75	0.18 ± 0.05	0.40 ± 0.26	9.41 ± 0.91	14.40 ± 0.77	48.17 ± 2.56
6 g/kg	3.85 ± 0.35	3.07 ± 0.62	0.20 ± 0.07	0.58 ± 0.33	9.68 ± 1.10	13.55 ± 1.25	48.33 ± 2.66
12 g/kg	3.44 ± 0.73	2.95 ± 0.70	0.14 ± 0.03	0.34 ± 0.13	9.40 ± 1.57	14.13 ± 2.28	48.17 ± 2.32

Note: Results are expressed as mean ± SD (*n* = 6). WBC means white blood cell count, LYM means lymphocyte, MON means monocytes, NEU means neutrophils, RBC means red blood cells, HGB means hemoglobin, and MCV means mean corpuscular volume.

**Table 9:** Effect on biochemical indicators of the FZBSP.

Groups	ALP U/L	ALT U/L	BUN mmol/L	GLU mmol/L	TP g/L
CON	253.67 ± 5.89	39.83 ± 1.47	10.50 ± 0.34	3.62 ± 0.13	56.50 ± 2.88
1.5 g/kg	251.33 ± 6.06	40.83 ± 1.33	10.40 ± 0.28	3.48 ± 0.21	55.00 ± 2.83
3 g/kg	251.83 ± 8.23	39.33 ± 1.03	10.28 ± 0.49	3.25 ± 0.26	55.33 ± 1.37
6 g/kg	253.83 ± 4.36	39.67 ± 1.75	10.67 ± 0.33	3.57 ± 0.23	55.50 ± 2.88
12 g/kg	252.17 ± 4.17	39.17 ± 1.17	10.55 ± 0.44	3.08 ± 0.49	54.83 ± 2.14

Note: Results are expressed as mean ± SD (*n* = 6). ALP means alkaline phosphatase, ALT means alanine aminotransferase, BUN means blood urea nitrogen, GLU means glucose, and TP means total protein.

## Data Availability

The original contributions presented in the study are included in the article; further inquiries can be directed to the corresponding authors.
